# Scalar Diversity, Negative Strengthening, and Adjectival Semantics

**DOI:** 10.3389/fpsyg.2018.01659

**Published:** 2018-09-12

**Authors:** Nicole Gotzner, Stephanie Solt, Anton Benz

**Affiliations:** ^1^Leibniz-Centre General Linguistics, Berlin, Germany; ^2^Department for German Language and Linguistics, Humboldt-University, Berlin, Germany

**Keywords:** scalar implicature, scalar diversity, scale structure, gradable adjectives, negative strengthening, negation

## Abstract

Previous research has demonstrated great variability in the rates of scalar inferences across different triggers (Doran et al., 2009; van Tiel et al., 2016). In the current study, we show that variation is more systematic than previously thought. In particular, we present experimental evidence suggesting that endorsements of scalar implicatures (i) are anti-correlated with the degree of negative strengthening of the stronger scale-mate (e.g., whether *John is not stunning* is interpreted as conveying that John is rather ugly) and (ii) are affected by the scale structure and the underlying scalar semantics of gradable adjectives (in particular boundedness, polarity, and adjectival extremeness). Overall, our research suggests that scale structure should be taken into account in theories of implicature.

## 1. Introduction

According to a tacit assumption in the theoretical and experimental literature, scalar implicature is based on a single mechanism, and the behavior of one scale generalizes to the whole family of scales (van Tiel et al., 2016). Contrary to this so-called uniformity assumption, experimental research has demonstrated great variability in the rates of scalar inferences across different triggers, in part being explained by factors such as grammatical category, boundedness, and semantic distance between scale-mates (Doran et al., 2009, 2012; van Tiel et al., 2016). These experimental studies have provided evidence that gradable adjectives in particular tend to yield low rates of scalar implicature (e.g., see the conclusions in Doran et al., 2012; Beltrama and Xiang, 2013).

In the current study, we focus on scalar implicatures and a specific kind of manner implicature triggered by negated adjectives, referred to as negative strengthening (Horn, 1989). Negative strengthening describes the phenomenon by which an utterance such as *John is not brilliant* receives a stronger interpretation than its semantic meaning, for example that John is “rather stupid” or less than intelligent. This interpretation is derived as a Manner or I implicature (Horn, 1989; Levinson, 2000) or explained as a blocking phenomenon in optimality theory (Blutner, 2000; Krifka, 2007). Theories agree that scalar implicature and negative strengthening are two different kinds of implicature, which arise from distinct conversational principles, the Q and R principles, respectively (Horn, 1989; Levinson, 2000). These principles are assumed to govern each other; therefore an interaction between the two kinds of pragmatic strengthening is expected (see Krifka, 2007)^1^.

In this paper, we present two experimental studies investigating the interaction of scalar implicature and negative strengthening in different types of gradable adjectives. We will show that for some adjectives the effect of scalar implicature may be masked by the presence of negative strengthening. Further, we provide evidence that the scale structure associated with the semantics of gradable adjectives affects the likelihood with which a scalar implicature and negative strengthening are derived.

In the following, we discuss how scalar implicature and negative strengthening affect the interpretation of gradable adjectives and we review previous studies on scalar diversity. Then, we present the results of two experiments and discuss the relevance of the findings to the phenomenon of scalar diversity.

### 1.1. Interaction between scalar implicature and negative strengthening

**Figure 1:**
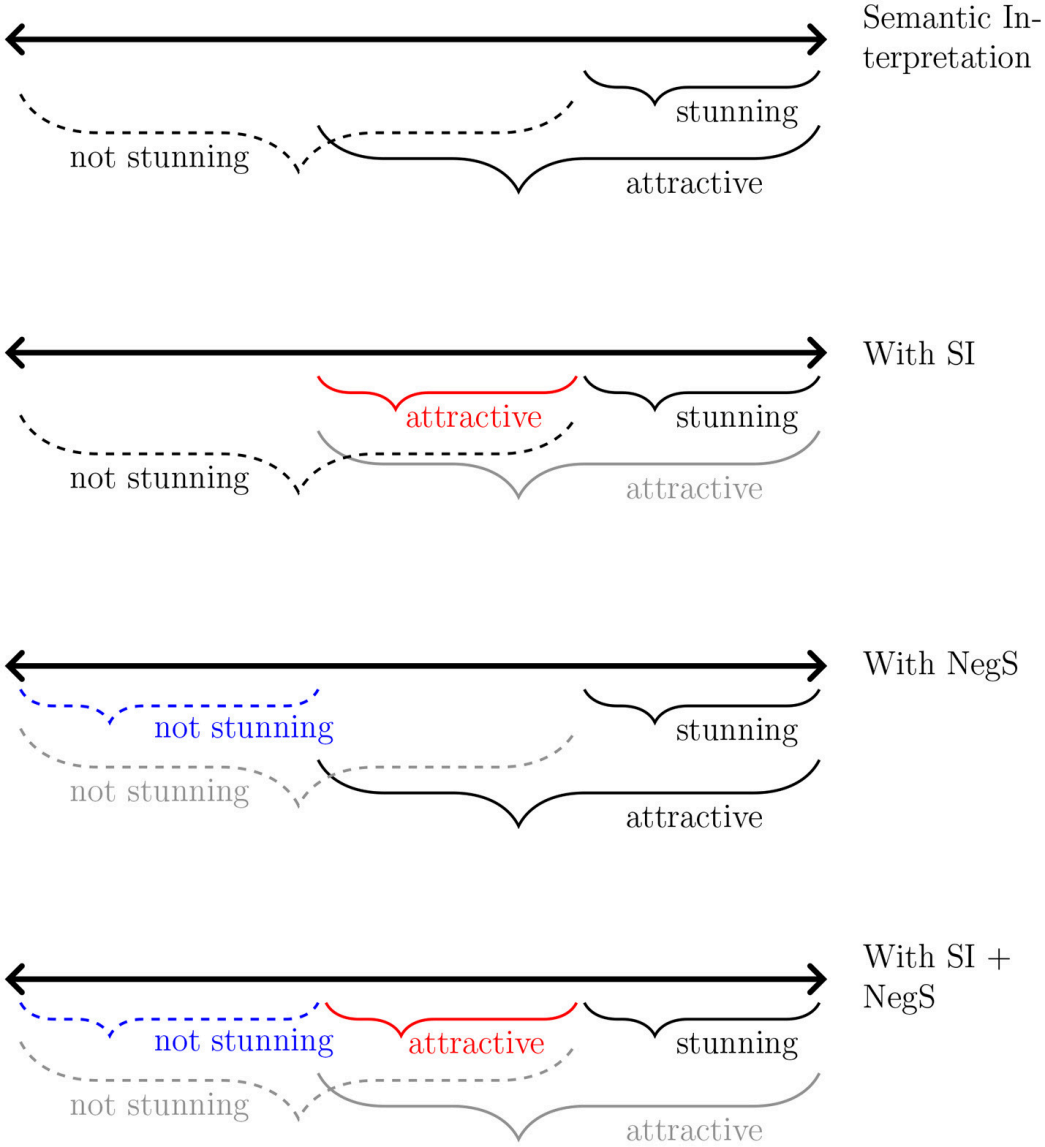
Semantic interpretation of *attractive, stunning*, and *not stunning*. The effect of scalar implicature on *attractive* is represented in red and that of negative strengthening on *not stunning* is represented in blue.

In this section, we explore the interplay of different kinds of implicatures and the interpretations they lead to. To see the effect of semantic interpretation and pragmatic inference on statements involving weak and strong scalar terms, consider the scale of attractiveness depicted in Figure 1. The first line represents the semantic interpretation of an utterance like *John is attractive*, which is compatible with the stronger alternative statement *John is stunning*. The second line depicts the effect of scalar implicature, namely that the stronger statement is implicated not to obtain, such that the weaker term *attractive* is understood to apply only to the more restricted range of being “attractive but not stunning.” This implicature is based on the maxim of quantity, since the two scale-mates stand in an entailment relationship with each other and the stronger term is more informative than its weaker scale-mate.

Now consider the case where the strong scalar term appears under negation. As shown in the first line of Figure 1, *John is not stunning* entails on its semantic meaning merely that John is something less than stunning (i.e., it leaves open whether he might be attractive, merely average looking, or downright ugly). The third line of Figure 1 shows the effect of negative strengthening on the stronger scalar item: If the statement *John is not stunning* is negatively strengthened, the resulting meaning is inconsistent with John being attractive (i.e., it is consistent with him being average looking or ugly). In a similar vein, an asymmetry between positive and negative expressions has been pointed out (Brown and Levinson, 1987; Horn, 1989; Ruytenbeek et al., 2017). For example, while an utterance like *John is not tall* is often interpreted as John being rather short, the statement with the antonym (*John is not short*) is unlikely to be strengthened in order to convey that John is rather tall. The assumption is that the positive adjective denotes a desirable property in contrast to its antonym, relating the negative asymmetry to euphemism and understatement (Brown and Levinson, 1987; Horn, 1989; Krifka, 2007). However, it is easy to find counterexamples to this asymmetry and it is unclear which notion of polarity is the relevant one (e.g., emotional valency vs. negative morphology) or which adjective constitutes the positive form (see especially Ruytenbeek et al., 2017). We will return this issue below.

As the fourth line of Figure 1 shows, when both apply, scalar implicature and negative strengthening divide up the range of possible interpretations categorically. However, as we will see this pattern may only hold in the case of certain types of gradable adjectives.

Note also that there is a possibility not reflected in Figure 1, namely that negated strong expressions receive a so-called scale-reversal or indirect implicature (e.g., see Horn, 1989; Chierchia, 2004; Romoli, 2012; Gotzner and Romoli, 2017). A classic example is that the utterance *John did not eat all of the cookies* implicates that he ate some of them. This scale reversal implicature occurs when the strong scale-mate appears under negation and it is assumed to arise by the same mechanism as (direct) scalar implicature. The crucial difference is that negation reverses entailment relationships and therefore the *not all* is replaced with the alternative *not some*. Thus, the negation of the stronger alternative *not some* leads to the inference that John ate some of the cookies.

While scale reversal implicature and negative strengthening are based on different types of conversational principles, they stand in direct competition with each other. When a sentence contains a negated scalar term, scale reversal leads to the endorsement of the weaker scale-mate while negative strengthening excludes the weaker scale-mate. Thus, hearers may be inclined to take into account both considerations of informativeness and manner when deciding whether the weaker term applies (that is, whether the speaker wanted to convey that the weaker term applies or not).

### 1.2. Experimental evidence for scalar diversity

There have been several experiments investigating the likelihood with which different scalar terms trigger a scalar implicature. Doran et al. (2009, 2012) investigated the availability of such inferences across a range of scale types, using a truth value judgment task in which participants were presented with a statement containing a weak scalar term and a fact which would support the use of a stronger term, and were asked to indicate whether a literally minded character “Literal Lucy” would say the sentence was true or false given that fact. The results showed that upper-bounding inferences were less likely to arise in the case of gradable adjectives than for quantifiers, cardinal numerals or rank orderings. Furthermore, only in the case of adjectives did the explicit mention of stronger scale-mate alternatives have the effect of increasing the rate of implicatures.

In an experiment employing a felicity-judgment task, Beltrama and Xiang (2013) similarly found evidence that adjectives behave differently from modal expressions with respect to the triggering of scalar implicatures, and furthermore that adjectives themselves differ in the extent to which they give rise to implicatures. Specifically, it was found that weak positive adjectives (e.g., *decent*) tend to implicate the negation of the corresponding middle and extreme adjectives (e.g., *good, excellent*), but middle adjectives do not implicate the negation of the extreme adjective. No such difference was found for modal expressions. The authors suggest several possible explanations for their findings, including relative semantic distance between scale-mates, the particular semantic properties of extreme adjectives, and the unbounded nature of adjectival measurement scales as opposed to the bounded nature of modal scales (see also Simons and Warren, 2018 for further evidence on the role of boundedness and relevant discussion).

A more extensive and fine-grained investigation of potential variability in scalar implicature rates is that of van Tiel et al. (2016), who investigated 43 weak/strong scalar pairs from a variety of grammatical categories, including adjectives, determiners, verbs, and adverbs. In their experiment, participants were presented with statements involving a weak scalar term and were asked whether they would infer the negation of a stronger scale-mate, for example whether the statement in (1) licenses the scalar inference in (2).

1. John is attractive
2. John is not stunning

The main finding of the van Tiel et al. (2016) study was a high variability in endorsement rates of the scalar inference across triggering expressions. For example, while few participants endorsed the potential scalar inference in (2) triggered by the weak term *attractive*, almost all participants endorsed the scalar inference associated with *some*. The authors also systematically investigated a range of factors that could account for scalar diversity. As potential predictors of variability in inference rates, van Tiel et al. (2016) probed the semantic distance between the weaker and stronger term, their association strength, the availability of the stronger term, its relative frequency, as well as the presence of an upper bound on the underlying measurement scale. The only significant predictors were upper boundedness and semantic distance (as measured by a rating of the perceived difference in strength between the statements involving the weaker and stronger term). But a large proportion of the overall variance in inference rates remained unexplained by any of the potential predictors investigated. Overall, the study by van Tiel et al. (2016) has been taken as evidence refuting the uniformity hypothesis.

Benz et al. (2018a) revisited the methodology and findings of van Tiel et al. (2016), raising the possibility of a confound due to the presence of negative strengthening. As described above, the experimental materials in van Tiel et al.'s study included negated stronger scale-mates, which as discussed above may give rise to negative strengthening. Consider our example (2) from above. The utterance *John is not stunning* may be strengthened to convey that John is rather ugly, which is incompatible with the semantic meaning of *attractive*. This could have the effect of masking the presence of scalar implicature. That is, participants in van Tiel et al.'s task may have derived a scalar implicature for the weaker scale-mate but decided nonetheless to respond with *No* because the strengthened reading of the stronger scale-mate stood in conflict with their interpretation of the implicature-modified weaker term. If participants interpreted *John is attractive* as “attractive but not stunning” but *John is not stunning* in the conclusion sentence is interpreted as John being rather ugly, then the *No*-answer is simply based on the presence of negative strengthening—not the absence of scalar implicature for the weaker scale-mate. To be clear, this additional pragmatic strengthening comes into play because the conclusion sentence contains a negated strong scalar term and this affects the interpretation of the original statement of interest.

Benz et al. (2018a) carried out an experiment using the same set of materials used by van Tiel et al. in which participants saw a statement involving the negation of the stronger scale-mate and were asked whether the negation of the weaker term followed, as a measure of negative strengthening. For example, participants were asked whether an utterance like (2) *John is not stunning* suggests that John is not attractive. It was found that that endorsements of scalar implicature were anti-correlated with the degree of negative strengthening of the stronger scale-mate. The study thus provided evidence for the assumption that participants did not endorse the scalar implicature with certain triggers because they negatively strengthened the stronger term. Further, the authors presented additional analyses showing that the data by van Tiel et al. are consistent with a modified version of the uniformity assumption, once negative strengthening is taken into account.

The above study also found a potential explanatory role for factors including semantic distance and boundedness (i.e., the factors identified by van Tiel et al. as being significant predictors of scalar implicature rates). However, Benz et al. note that there was high degree of overlap between potential predictors in the stimulus material (e.g., between boundedness and grammatical category), making it difficult to draw firm conclusions as to the source of the observed effects.

### 1.3. Scale structure, adjective meaning, and implicature

The existing body of experimental research on scalar diversity has provided evidence that adjectives behave differently from other sorts of scalar items when it comes to the derivation of scalar implicatures. But it is less clear why this should be, or indeed the extent to which it is the case for all adjectival pairs or only certain salient subclasses.

The results of these previous studies also suggest that properties of the underlying measurement scales lexicalized by gradable adjectives (and perhaps items of other classes) play a role in determining the frequency at which they give rise to scalar implicatures^2^. Here too, there are a number of questions that remain to be explored.

As noted above, one factor found to be a significant predictor of scalar implicature rates is boundedness, namely whether or not the stronger member of a lexical scale denotes a scalar endpoint of some sort. The notion of boundedness is familiar from the literature on the semantics of gradable adjectives (see especially Kennedy and McNally, 2005; Kennedy, 2007), where it has been shown to explain a diverse range of combinatorial and interpretive phenomena. The central observation is that the measurement scales lexicalized by gradable adjectives may differ as to whether they have maximum and/or minimum points. This is claimed in particular to determine the interpretation of the adjective in its unmodified “positive” form. If the scale is lower closed, the corresponding adjective has an existential minimum standard (to be dirty is to have some amount of dirt); if it is upper closed, the adjective has a maximum standard (to be clean is to have a maximal degree of cleanness). Both of these are known as absolute interpretations. By contrast, if the scale is open on both ends, the adjective has a context-dependent relative standard (what counts as tall depends on the context and the sorts of entities under consideration).

Importantly, the bounded adjectival cases in van Tiel et al.'s study do not correspond to the class of maximum standard gradable adjectives from the adjectival literature, but rather involve a somewhat heterogeneous mix of measurement scale structures and adjective meanings. In some pairs, the weaker term is a relative gradable adjective, while the stronger term is a non-gradable adjective denoting the scalar endpoint; in the theory of Kennedy & McNally, this point is actually not part of the measurement scale lexicalized by the weaker adjective. Furthermore, in some such pairs (e.g., *cheap/free*) the stronger term denotes a scalar “zero” point, i.e., the complete absence of some property, whereas in others (e.g., *good/perfect*) it denotes some maximum point. Finally in other cases, the measurement scale itself is plausibly closed on both ends; the weaker term has a minimum standard existential interpretation while the stronger term is maximum-denoting (e.g., *allowed/obligatory, possible/certain*). In fact, van Tiel et al.'s experimental materials contain no “classic” examples of maximum-standard gradable adjectives. This gap makes it difficult to clearly diagnose the scope of the boundedness effect, and its source.

If the interpretation of the stronger scale-mate (namely whether or not it is endpoint-denoting) plays a role in determining the frequency at which scalar implicatures will arise, we might hypothesize that the interpretation of the weaker scale-mate will likewise play a role. And indeed, Benz et al. (2018a) find a difference between those lexical scales in which the weaker scale-mate has a greater-than-minimum or existential interpretation (L scales) and those where it invokes a mid-scale standard (M scales): only in the latter case does the negative correlation between scalar implicature and negative strengthening obtain. They note however that the set of M scales in the original materials from van Tiel et al. largely overlaps with the set of adjectival scales, while the L scales involve primarily items of other grammatical categories such as quantifiers and verbs; thus the potential role of this aspect of scale structure cannot be separated from that of grammatical category.

Put in different terms, the adjectival scales investigated to date in the scalar diversity literature largely involve relative gradable adjectives as the potential implicature trigger. Few minimum standard adjectives have been tested, and thus it is not yet known how this subclass will pattern with respect to the two types of implicature investigated here. One previous investigation by Leffel et al. (in press) showed that lower bounded adjectives like *late* and relative adjectives like *tall* are interpreted differently in the “not very” construction. In particular, Leffel et al. (in press) found that the utterance *John was not very late* yielded an inference to the positive form (that John was late) while the utterance *John is not very tall* was interpreted as meaning that John is not tall (with negative strengthening).

Finally, even among the relative gradable adjective pairs that make up the majority of the adjectival scales tested to date, there is diversity in the structures of the underlying measurement scales, and in how the individual members of the pairs relate to those scales. In particular, the items tested to date include both positive adjectives (e.g., *big/enormous*) and negative adjectives (e.g., *small/tiny*). As discussed above, positive vs. negative polarity has been argued to be relevant to the likelihood of negative strengthening, and we thus might expect it to play a role for scalar implicature too; but this has not yet been systematically investigated. Furthermore, in many of the pairs tested (e.g., *good/excellent*) the weaker term is a basic-level term while the stronger one is an extreme adjective (Morzycki, 2012); but in several cases (e.g., *adequate/good*), the weaker term describes something like a moderate degree of the property in question, while the stronger one is the basic-level term. Also as discussed earlier, Beltrama and Xiang (2013) found evidence for a lower level of scalar implicatures to the negation of an extreme adjective than to the negation of a mid-scale adjective; but the role of this factor as a potential predictor has not been taken into consideration in the more recent literature on scalar diversity.

In light of the issues discussed above, further research into the potential predictors of scalar diversity is needed, particularly as it pertains to adjectival scales (see also a recent commentary by McNally, 2017).

### 1.4. Goals of the current study

The current study investigates the interplay of scalar implicature and negative strengthening for a broader and more balanced range of scalar adjectives. We have decided to focus on adjectival scales for several reasons. First, adjectives constituted the majority of items in van Tiel et al. (2016), and we wanted to further evaluate the claim that they generate low rates rates of implicature (Doran et al., 2012; Beltrama and Xiang, 2013). Second, the semantics of the class of gradable adjectives is well described and it is possible to tease apart factors related to the structure of the underlying measurement scales. Third, adjectives belong to the set of open class terms, thereby providing a rich set of items. In contrast to previous work, we include a much more varied set of adjectival scales, and code these on a fuller set of scalar properties that we hypothesize to be relevant to the availability of pragmatic inferences.

The first goal of our study is to determine whether the (anti-)correlation between scalar implicature and negative strengthening found by Benz et al. (2018a) for van Tiel et al.'s original items is also replicated for a wider range of adjectival scales. The second is to provide further insight into the predictors of variability in the rates of these inferences, with a focus on examining the role of factors relating to the underlying structure of the scales lexicalized by gradable adjectives.

## 2. Experiments

### 2.1. Methods

#### 2.1.1. Participants

Participants with US IP addresses were recruited on Amazon's Mechanical Turk platform and were further screened for native language. In total, 220 native English speakers (mean age: 37.4, 95 female, 121 male, 4 gender information not given) took part in the study.

The experiments were conducted in accordance with the ethics policy of the Deutsche Forschungsgemeinschaft (DFG) under approval of grant Nr. BE 4348/4-1. Since the study involved a healthy adult population, no ethics consent was required according to institution's guidelines and national regulations. Participant's consent was obtained by virtue of survey completion and their data were fully anonymized.

#### 2.1.2. Materials

##### 2.1.2.1. Items

We created a set of 70 adjective pairs with weak and strong scale-mates^3^. We took all adjective pairs from the van Tiel et al. study (32) and added a further set of 38 adjective pairs to balance factors related to the scale structure of the adjectives. In particular, we added further absolute gradable adjectives (minimum standard and maximum standard), as well as more pairs where the stronger scale-mate is non-extreme. Tables A1, A2 in Appendix presents a list of all 70 adjective pairs. These were embedded in 7 separate tasks administered to 40 participants each (except for the politeness ratings which only involved 20 participants).

##### 2.1.2.2. Main tasks

**Figure 2:**
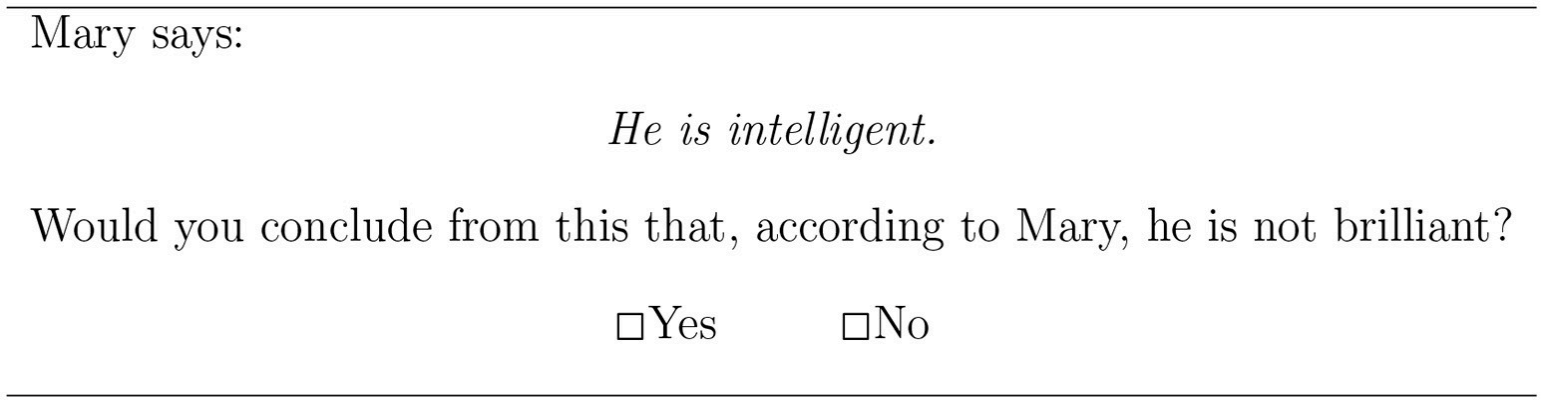
Sample item of the scalar implicature task (based on van Tiel et al., 2016).

The two main tasks employed the paradigm from van Tiel et al. (2016). Participants are presented with a scenario involving two characters, Mary and John, who make a series of statements. Their task is to decide whether a strengthened interpretation follows from a given statement. In the first task, participants were presented with the weaker term and had to indicate whether they endorse the negation of the stronger term, i.e., the scalar implicature. For example, Mary said: *John is intelligent* and participants were asked whether, according to Mary, John is not brilliant. Figure 2 presents a sample display participants saw.

**Figure 3:**
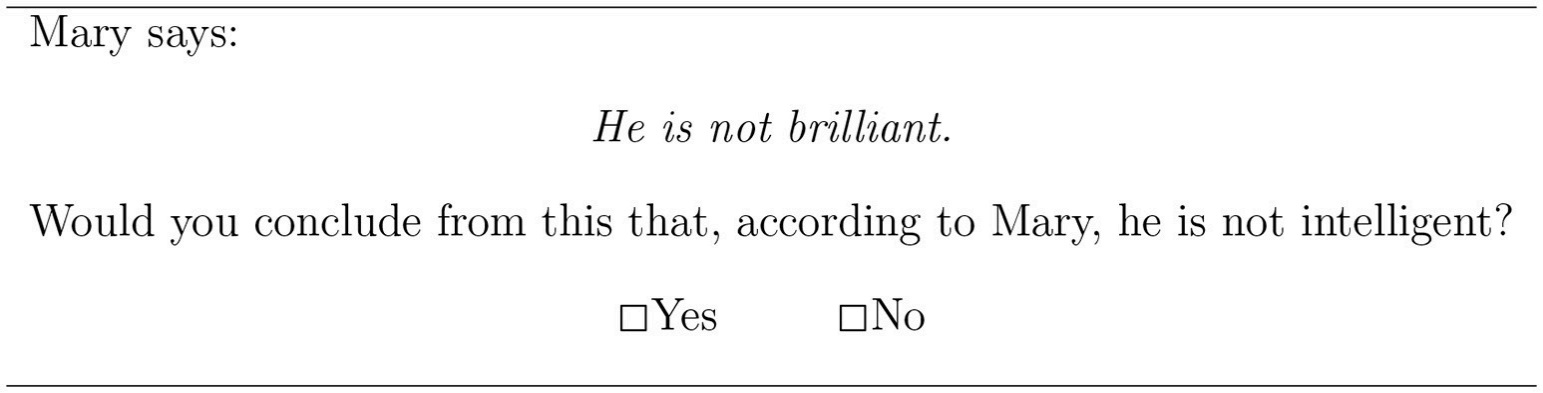
Sample item of the negative strengthening task.

In the second main task, participants were asked whether the negation of the stronger term suggests the negation of the weaker term. For example, participants saw the statement *John is not brilliant* and were asked whether they conclude that John is not intelligent. The latter task is a measure of negative strengthening of the stronger scale-mate. Figure 3 gives an example.

Two survey versions of the main tasks were created and administered to 20 participants each.

##### 2.1.2.3. Additional rating experiments

Additionally, we collected a variety of measures based on the methodology of van Tiel et al. (2016). First, we had participants rate the **semantic distance** between the statements involving the weaker and stronger scale-mate. In this task, participants were presented with a pair of two statements, one with the weak term and one with the strong term. Participants were asked how much stronger the second statement is compared to the first one. They gave their answer on a 1–7 point Likert scale with 7 indicating that the second statement is much stronger and 1 that the statements are equally strong.

Second, we administered a **cloze task** to another set of participants, in order to measure association strength between the weaker and stronger terms. We used the open version of the task by van Tiel et al. in which participants had to mention three words that come into their mind upon seeing the statement with the weaker term. Participants' responses were then coded for the frequency of mentioning the stronger scale-mate. We employed a strict scheme for coding the responses, only taking into account exact mentions of the stronger scale-mate^4^.

Finally, participants also rated the kindness/politeness of statements involving the weaker term, the stronger term and the negated stronger term. Here we used the methodology of a previous study by Bonnefon et al. (2009). In each task, participants rated how nice the respective statement was on a 1–7 point scale with 7 indicating that the statement was very nice. This rating was included because negative strengthening has been discussed with respect to politeness considerations.

Table 1 presents an overview of the different tasks we ran. All of these tasks, except for the politeness rating, were administered in two survey versions with different orders to 20 participants each.

**Table 1:** Overview of tasks.

**Label**	**Task**	**Intended measure**
Main task SI	Inference judgment (yes\no)	Scalar implicature
Main task NegS	Inference judgment (yes\no)	Negative strengthening
Semantic distance	Strength rating (1–7 scale)	Scale distinctness
Cloze task	Free word production	Association strength
Politeness weak	Kindness rating (1–7 scale)	Weak statement
Politeness strong	Kindness rating (1–7 scale)	Strong statement
Politeness “not” strong	Kindness rating (1–7 scale)	Negated strong statement

##### 2.1.2.4. Annotation

In addition to the measures presented above, we annotated each pair on a range of scale-related properties, specifically the **boundedness** and **extremeness** of the stronger scale-mate, the **standard type** of the weaker scale-mate (minimum, relative, or maximum), and the **polarity** of the scale as a whole (positive or negtive).

In making these annotations, the following diagnostics were used: A pair was coded as **upper bounded** if the stronger member of the pair denotes a scalar endpoint, as evidenced by compatibility with endpoint-oriented modifiers such as *almost, completely*, and *100 percent* (e.g., *completely clean* vs. ??*completely tall*). A pair was classified as **extreme** if the stronger member of the pair patterns as extreme using Morzycki's [2012] test of compatibility with extreme adjectival modifiers such as *downright* and *flat-out* (e.g., *downright excellent* vs. ??*downright good*). Following the diagnostics of Kennedy and McNally (2005) and Kennedy (2007), a pair's weaker member was classified as having a **minimum** standard if is compatible with low-degree modifiers such as *slightly* and *a bit* (e.g., *slightly wet* vs. ??*slightly tall*), and if its negation entails a zero degree of the property in question; it was coded as having a **maximum** standard if it passes the tests for upper-boundedness described above, or shows other evidence of endpoint-orientation; and it was classified as **relative** otherwise. Note that pairs with a maximum-standard weaker scale-mate necessarily have a bounded (endpoint-denoting) stronger scale-mate, and represent cases of variation in the precision at which the standard is interpreted (e.g., *clean/spottless*). This will be relevant below.

Adjectival **polarity** proves to be the most complicated dimension to annotate. As discussed in Ruytenbeek et al. (2017), there are multiple notions of adjectival polarity, including morphological, dimensional, evaluative, and markedness-based ones, and individual tests do not apply equally well to all antonym pairs. We therefore followed those authors in implementing a step-wise classification, in which a series of tests were applied in sequence, as follows: (i) If the weaker member of the pair contains a negative morpheme, that pair was classified as negative. (ii) If the pair is associated with a quantitatively measurable dimension, the adjective pairs associated with higher measurement values were classified as positive and those associated with lower measurement values were classified as negative, based on acceptability in the frame “something with a larger (smaller) number/amount of x is *Adj-er*”. Note that this test applies both to adjectives traditionally considered dimensional (e.g., *tall/short*: “something with a larger (smaller) number of inches of height is *taller (shorter)*”) as well as to those with more complicated relations to measurable dimensions (e.g., *dirty/clean*: “something with a larger (smaller) amount of dirt is *dirtier (cleaner)*”). (iii) For adjectives expressing value or taste judgments, an evaluative notion of polarity (“good” vs. “bad”) was applied; (iv) Tests (i)–(iii) left 9 pairs still unclassified (*damaged/broken, faulty/non-functional, sleepy/asleep, light/white, dark/black, special/unique, calm/unflappable, tired/exhausted, hungry/starving*). These were annotated for polarity based on the authors' judgments. Note finally that this classification procedure identified some cases of conflict between dimensional and evaluative notions of polarity (e.g., *dirty* is dimensionally positive but arguably evaluatively negative). On account of our overall focus on the role of scale structural factors, we chose to prioritize the dimensional sense.

We also extracted the frequency of the weaker term, the stronger term and the negated stronger term from the Corpus of Contemporary American English (Davies, 2008). We calculated the relative frequency of the weaker and stronger term taking the logarithm of the frequency of the weaker divided by the stronger term (to make up for skewness of the distribution, see van Tiel et al., 2016). For negative strengthening, we took the logarithm of the frequency of the negated stronger term divided by that of the simple stronger term.

### 2.2. Results

#### 2.2.1. Results of main tasks (SI and Negs)

**Figure 4:**
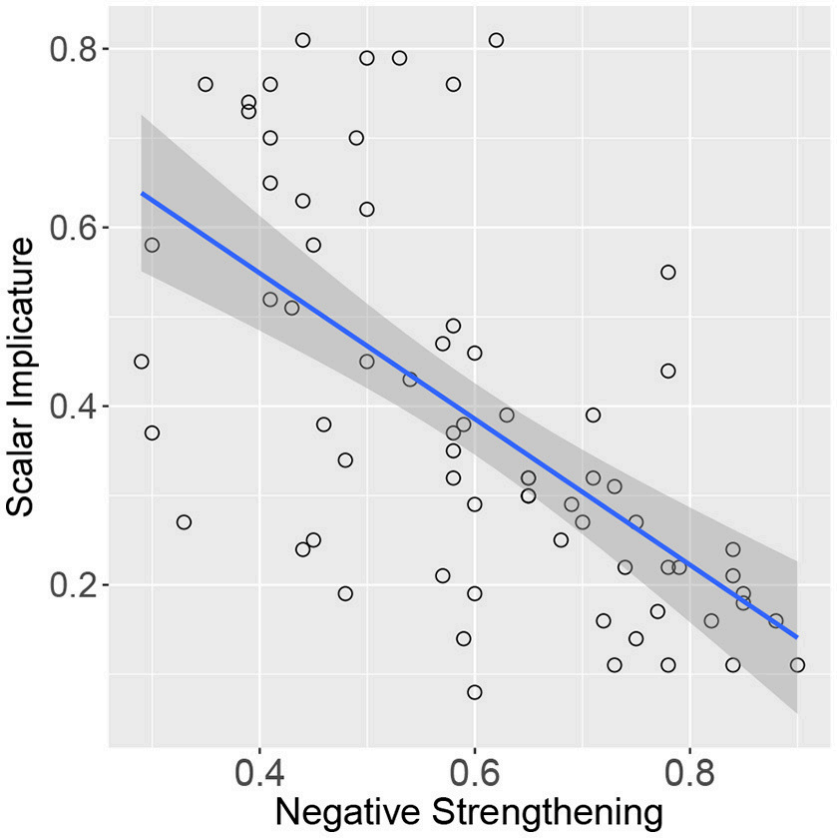
Correlation between endorsements in the scalar implicature and negative strengthening task (proportion of YES responses).

Table 2 presents a sample of adjectives with different scale structures and their respective endorsement rates in the scalar implicature (SI) and negative strengthening (NegS) tasks. The results for all scales are presented in Tables A1, A2 in the Appendix. A Pearson's correlation test revealed that the two ratings were anti-correlated (*r* = −0.62, *p* < 0.0001, see Figure 4). That is, the more likely participants applied negative strengthening to the stronger scale-mate, the less likely they were to endorse the scalar implicature.

**Table 2:** Example scales and their respective endorsement rates in the scalar implicature (SI) and negative strengthening (NegS) task.

**Weak/strong term**	**Scale structure**	**SI**	**NegS**
Cheap/free	Bounded rel neg non-extreme	0.76	0.41
Possible/certain	bounded min pos non-extreme	0.58	0.3
Clean/spotless	Bounded max neg extreme	0.27	0.75
Wet/soaked	Unbounded min pos extreme	0.24	0.44
Large/gigantic	Unbounded rel pos extreme	0.22	0.74
Scared/petrified	Unbounded rel neg extreme	0.14	0.75

#### 2.2.2. Predicting variability

We first calculated the mean values for each adjective pair in the seven different tasks. Then, we fit two linear regression models involving all predictors outlined above for the scalar implicature and the negative strengthening tasks respectively (see Table 3). The regression analysis showed that endorsements of the scalar implicature were higher for upper bounded scales (*p* < 0.01), more distant scale-mates (*p* < 0.0001), and higher for negative compared to positive scales (*p* < 0.05). Conversely, extreme adjectives yielded lower endorsement rates compared to non-extreme ones (*p* < 0.0001) and maximum standard weaker scale-mates lower rates than relative terms (*p* < 0.01)^5^. The multiple *R*^2^ of the SI model was 0.62 and the amount of explained variance for each predictor is listed in Table 3A.

**Table 3:** Predictors of endorsements in **(A)** the scalar implicature and **(B)** negative strengthening task.

	**Estimate**	**SE**	***t*-value**	***p*-value**	***R*^2^**
**(A) SI**					
(Intercept)	−0.295	0.190	−1.547		
Weak min	−0.024	0.049	−0.495	0.623	
Weak max	−0.208	0.079	−2.652	0.010	0.060
Upper bounded	0.140	0.049	2.840	0.006	0.117
Semantic distance	0.132	0.028	4.763	0.000	0.136
Polarity neg	0.088	0.042	2.103	0.040	0.047
Extremeness	−0.206	0.052	−3.963	0.000	0.165
Politeness weak	0.017	0.034	0.513	0.610	0.004
Politeness strong	0.002	0.021	0.108	0.914	0.004
Cloze probability	−0.370	0.242	−1.526	0.132	0.069
Relative frequency	−0.024	0.019	−1.233	0.223	0.021
**(B) NegS**					
(Intercept)	1.276	0.316	4.038		
Weak min	−0.040	0.044	−0.905	0.370	
Weak max	0.146	0.069	2.121	0.038	0.081
Upper bounded	−0.073	0.044	−1.644	0.106	0.056
Semantic distance	−0.105	0.025	−4.151	0.000	0.184
Polarity neg	0.012	0.037	0.320	0.750	0.003
Extremeness	0.129	0.042	3.048	0.004	0.085
Politeness weak	−0.022	0.024	−0.930	0.357	0.008
Politeness not strong	−0.036	0.044	−0.833	0.408	0.011
Cloze probability	0.012	0.033	0.367	0.715	0.022
Relative frequency	0.263	0.216	1.219	0.228	0.071

The negative strengthening task showed the opposite pattern with lower endorsement rates for more distant scale-mates (*p* < 0.0001) and higher rates for extreme adjectives (*p* < 0.01). The negative strengthening rates were higher for maximum standard weaker scale-mates compared to relative ones (*p* < 0.05)^6^. The multiple *R*^2^ of the NegS model was 0.52 and the amount of explained variance for each predictor is listed in Table 3B.

Finally, we assessed the effect of adding NegS rates as a predictor in the model for the SI task. The original model had an *R*^2^ of 0.62 and the new model with NegS as a predictor had a multiple *R*^2^ of 0.66; this improved fit was found be to be significant (model comparison test with the anova function: *p* < 0.05). The original factors extremeness, polarity and semantic distance remained as significant predictors in the new model but the difference between relative and maximum standard weaker scale-mates was marginal (*p* = 0.07). The results of the model are presented in Table 4.

**Table 4:** Model for endorsements in the scalar implicature with negative strengthening task as an additional predictor.

	**Estimate**	**SE**	***t-value***	***p-value***	***R*^2^**
(Intercept)	0.091	0.247	0.369	0.713	
NegS	−0.339	0.145	−2.340	0.023	0.189
Weak min	−0.034	0.047	−0.719	0.475	
Weak max	−0.148	0.080	−1.856	0.068	0.043
Upper bounded	0.103	0.050	2.051	0.045	0.087
Semantic distance	0.097	0.031	3.170	0.002	0.086
Polarity neg	0.094	0.041	2.328	0.023	0.050
Extremeness	−0.171	0.052	−3.264	0.002	0.126
Politeness weak	0.007	0.033	0.223	0.825	0.003
Politeness strong	0.008	0.021	0.384	0.702	0.004
Cloze probability	−0.278	0.237	−1.172	0.246	0.050
Relative frequency	−0.019	0.019	−1.036	0.305	0.017

### 3. General discussion

#### 3.1. Summary of main findings

The current experiments showed that endorsements of scalar implicature are anti-correlated with the degree of negative strengthening of the stronger scale-mate. At the same time, we replicated the finding by van Tiel et al. (2016) that upper-bound denoting and semantically distant scale-mates yield higher endorsement rates in the scalar implicature task with our extended set of adjectival scales. Going beyond the latter study, we found that several additional factors related to the scale structure underlying the semantics of different adjective types predict variability, in particular polarity, adjectival extremeness, and the nature of the standard invoked by the weaker scale-mate.

In our negative strengthening task, extremeness, and semantic distance also had an impact on endorsement rates but these effects went in the opposite direction. That is, negative strengthening rates were lower the more distant the scale-mates and, in turn, higher for extreme adjectives. We further found an effect for maximum standard weaker terms compared to relative and minimum standard terms in the negative strengthening task.

Finally, we computed a model for the scalar implicature task that took into account all factors (including negative strengthening) and with these factors we were able to account for 66 % of the observed variance.

#### 3.2. Interaction between scalar implicature and negative strengthening

At the beginning of this paper, we discussed the possibility that negative strengthening could mask the presence of scalar implicature in van Tiel et al. (2016)'s task. This hypothesis is supported by the finding of an anti-correlation between endorsement rates in the scalar implicature task and the negative strengthening task. In addition, negative strengthening rates were a significant predictor of endorsement rates in the scalar implicature task (and explained variance in addition to other significant predictors such as extremeness, polarity, and semantic distance). These findings provide evidence that, for some scales, participants did not endorse the scalar implicature due to the application of negative strengthening to the negated stronger scale-mate.

Looking at the endorsement rates in the two tasks in comparison, however, there are some scales which received high negative strengthening rates as well as high scalar implicature rates. We therefore take our findings to indicate that negative strengthening is one among many factors which determines whether a scalar implicature is derived. This is also evident in the fact that scale structure factors such as boundedness, polarity, and extremeness remained significant predictors even when negative strengthening was taken into account. Further, for some scales, scalar implicature is robust and remains unaffected by negative strengthening while for other scales the propensity of triggering negative strengthening seems to be higher.

More generally, our findings corroborate the assumption that quantity and manner implicatures can both occur for the same pairs of lexical items. In other contexts, however the two might stand in competition with each other (see for example Levinson, 2000). Hence, our findings motivate further theoretical research into negative strengthening and how exactly different kinds of implicature are related to each other and how they interact in a specific context. In Gotzner et al. (in preparation), theoretical underpinnings of the attested interaction between scalar implicature and negative strengthening.

#### 3.3. Scale structure

We found that several factors related to scale structure had an effect on the rates at which the two kinds of inferences were generated. In what follows, we consider each of these in turn.

##### 3.3.1. Boundedness and the absolute/relative distinction

In the present study, we found that participants were more likely to endorse a scalar inference if the lexical scale of alternatives was upper bounded, meaning that the stronger scale-mate denotes an endpoint on some underlying measurement scale. Thus we replicated van Tiel et al.'s finding that boundedness is a significant predictor of scalar implicature rates. However, we also found that it is not all upper bounded lexical scales that behave this way. Specifically, we observed low rates of scalar implicature endorsement when the weaker scale-mate is itself a maximum standard gradable adjective, while the stronger term denotes that standard interpreted at a higher level of precision (e.g., *clean/spotless, dry/parched*). Thus it is not upper boundedness *per se* that is associated with higher implicature rates, but rather those lexical scales in which an endpoint-denoting stronger term stands in opposition to a minimum standard or relative standard weaker term.

van Tiel et al. (2016) discussed the boundedness effect in terms of scale distinctness, a broader concept that encompasses also semantic distance (which also had a significant effect; see below). That is, if the stronger scale-mate denotes an upper bound it is more clearly distinguishable from the weaker term, and therefore participants may be more likely to derive a scalar implicature. While we think that this characterization is compatible with our findings, we would also like to entertain the possibility that scale boundedness plays a more fundamental role in implicature computation, though perhaps in different ways for different sorts of adjectival pairs.

As discussed above, the literature on adjectival semantics (Kennedy and McNally, 2005; Kennedy, 2007) draws a distinction between two types of gradable adjectives: absolute gradable adjectives, which lexicalize measurement scales that are closed on one or both ends, with those endpoints providing a fixed standard of comparison for the adjective; and relative gradable adjectives, which lexicalize open scales, and thus have contextually determined standards. Psycholinguistic work has shown that listeners necessarily consider the standard value as part of the comprehension process of a sentence containing an absolute adjective (the “Obligatory Scale” hypothesis entertained by Frazier et al., 2008). It is plausible that similar factors might make scalar endpoints, and thus the adjectives that refer to them, particularly salient alternatives for the purposes of scalar implicature calculation.

Such an explanation holds potential in particular for adjectives such as *allowed/obligatory* and *possible/certain*, which arguably lexicalize totally closed scales. However, many of the upper-bounded adjectival pairs included in our study involved a relative gradable adjective as the weaker scale-mate (e.g., *cheap/free, scarce/unavailable, good/perfect*). In the adjectival literature, these are analyzed as lexicalizing totally open scales; the stronger term may then be analyzed as denoting a point that is actually not on the scale lexicalized by the weaker term. In these cases, we see it as possible that the use of the weaker scale-mate itself implies or even presupposes that the value described is on the non-endpoint portion of the scale, without any need for reference to a stronger potential alternative.

Factors relating to the lexical semantics of adjectives are also relevant in the case of unbounded scales of alternatives, that is, scales where the stronger scale-mate is not endpoint denoting. In most such pairs, the stronger term has a relative interpretation, according to which the standard is fixed contextually, with respect to the given comparison class (Solt, 2011; Solt and Gotzner, 2012). The values on the scale depend heavily on the noun that the adjective modifies and other contextual factors (see Rips and Turnbull, 1980 for psycholinguistic evidence that finding a standard for relative adjectives involves extra computation when the reference class is not mentioned).

For the computation of scalar implicature this may have the following consequences: (1) participants may not compute a scalar implicature to the negation of a stronger scale-mate with a relative interpretation because the stronger term does not stand in competition with the weaker term, i.e., because the stronger term might not come to mind in the same context or be relevant for the same comparison class.

Additionally, (2) relative adjectives may be less prone to implicature derivation because people have difficulty identifying the borderline for which the terms apply. Such a proposal has been made by Leffel et al. (in press), who formulated a constraint on implicatures such that they are not drawn if a borderline contradiction would be the result. Leffel et al. (in press) showed that lower bounded adjectives like *late* and relative adjectives like *tall* give rise to distinct inference patterns. For example, the utterance *John was not very late* yielded an inference to the positive form (that John was late) while the utterance *John is not very tall* was interpreted as meaning that John is not tall (with negative strengthening). Based on these data, Leffel and colleagues proposed a constraint according to which implicatures are not derived if they lead to a borderline contradiction. By the same token, we may hypothesize that participants in our study were reluctant to draw an inference from, say, *intelligent* to *not brilliant* or *big* to *not enormous* because they were uncertain as to where the scalar boundary for the stronger term lies.

Finally, while we found a difference in implicature rates between scales with endpoint-denoting and non-endpoint-denoting stronger scale-mates, and those with maximum standard vs. non-maximum-standard weaker scale-mates, there was perhaps surprisingly no difference between lexical scales with minimum-standard weaker terms and those with relative weaker terms (on either the scalar implicature task or the negative strengthening task). We see this as an issue requiring further investigation, in particular since the study by Leffel et al. (in press) found these two classes to behave differently with regards to a related variety of pragmatic inference.

##### 3.3.2. Extremeness

In our study, there was an additional effect related to scale structure that is relevant in this discussion. We found that extreme adjectives obtained lower implicature rates compared to non-extreme ones. If orientation toward the endpoint was the crucial factor in implicature computation, then extreme adjectives should yield higher implicature rates, contrary to what we have found. We assume that the effect of adjectival extremeness is of a different nature. Extreme adjectives have a particular semantics and they behave peculiarly in certain respects (see especially Morzycki, 2012 who entertains the view that extreme adjectives signal that the degree lies outside of the contextual range). Extreme adjectives may only be used in specific contexts and therefore again it might not arise as a competitor alternative out of the blue (see also Beltrama and Xiang, 2013). In turn, the use negated extreme adjectives may indicate that the situation is non-stereotypical thereby encouraging negative strengthening, as we have found in our negative strengthening experiment. This would be in line with the account of negative strengthening by Horn (1989) and Krifka (2007) according to which more complex expressions are used for less stereotypical instances. For example, the utterance *John is not tall* will tend to be used to describe cases that fall under the literal meaning of *short* (since *short* and *not tall* have the same literal meanings), but which are greater in height than the ones described by the utterance *John is short*.

##### 3.3.3. Semantic distance

**Figure 5:**
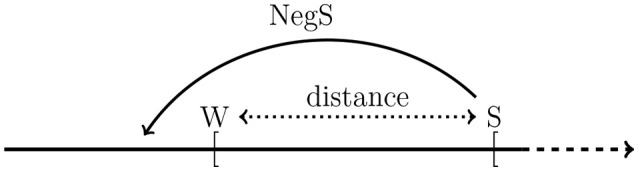
Semantic distance and negative strengthening. W and S are the lower bounds of the weak and strong scalar term, respectively, on an underlying measurement scale. As the distance increases, the more likely it becomes that an utterance of W implicates that S is excluded (SI), and the more difficult it becomes that the negation of S jumps over W into the region below W (NegS).

Negative strengthening and scalar implicature are differentially affected by semantic distance: as semantic distance between weak and strong scale-mates increases the SI–rate increases and the NegS–rate decreases. There is a suggestive explanation of this behavior if semantic distance is considered as distance between the lower bounds of the weak and the strong scale-mate on an underlying measurement scale, see Figure 5. The semantics of the weak term (W) always includes that of the strong term (S), however, the most likely value on a measurement scale that the speaker had in mind when producing W and S may be some distance apart. We may think of semantic distance as the distance between the lower bounds of the intervals defined by W and S. As the distance between the lower bounds increases, the more likely it becomes that the speaker means by saying W that S is not the case, and, hence, it becomes more and more likely that subjects answer that saying W implies not-S (SI), i.e., that a scalar implicature occurs. Negative strengthening (NegS) is explained as a *blocking* phenomenon (Horn, 1989; Levinson, 2000; Blutner, 2004; Krifka, 2007) which can be understood as a consequence of Horn's (1984) *principle of the division of pragmatic labor*, according to which a speaker who has a choice between a marked and an unmarked expression will prefer the unmarked one and, hence, signal by a choice of the marked expression that the unmarked one is not applicable. Hence, the existence of the unmarked expression *blocks* parts of the semantic meaning of the marked expression. In the case of scale mates (S,W) this means that if a speaker uses the marked expression not-S, then the existence of the unmarked W blocks not-S from having the meaning that could have been expressed by W. If the distance between W and S widens, W has to block a larger and larger interval on the underlying measurement scale, and it may become more and more improbable that W succeeds in doing this. As a result, the rate of negative strengthening will decrease with increasing semantic distance.

##### 3.3.4. The role of scale structure in implicature computation

Overall, we take our results to indicate that scale structure associated with the semantics of different adjectives systematically encourages or blocks certain inferences (see also Leffel et al., in press). We hypothesize that scale structure puts constraints on the range of potential values and thereby determines the alternatives used in implicature computation. Thus far, insights from the lexical semantics of scales have not been taken into account in the theory of implicature. Our investigation highlights the role of scale structure in pragmatic strengthening.

### 3.4. The role of polarity and politeness

As mentioned in the introduction, an asymmetry between positive and negative adjectives has been taken as evidence that negative strengthening is related to politeness considerations. Evidence for such an asymmetry between positive and negative adjectives was found in the experimental studies by Ruytenbeek et al. (2017) but previous experimental studies cited therein provided mixed results.

In the current study, we did not find any evidence that politeness ratings predicted variability in scalar implicature or negative strengthening rates. We did, however, find that polarity itself is an independent predictor of scalar implicature rates (though not of negative strengthening). Specifically, we found higher implicature rates with negative antonyms compared to positive ones.

Recall from section 3.1 that we chose to prioritize a dimensional notion of adjectival polarity, according to which the positive member of an antonym pair is the one that corresponds to a higher amount of some measurable property. As we noted above, this classification leads to some discrepancies with the evaluative notion of polarity. For example, according to the dimensional point of view the adjective *dirty* is the positive antonym (since it involves greater amounts of dirt), while *clean* is the the negative one (involving lesser amounts of dirt). In contrast, the evaluative notion of polarity would result in exactly the opposite classification since typically *clean* seems to be considered a desirable property. We also ran some additional models in which we restricted our analysis to the clear cut cases of the dimensional view of polarity and this analysis replicated the main results for the effect of polarity in the scalar implicature task (while again no such effect was present in the negative strengthening task).

In fact, the negative adjectives that yielded the highest levels of scalar implicatures in our study included many for which the stronger scale-mate denotes the complete absence of some quantity or property (e.g., *inaudible, extinct, free, unavailable*). We hypothesize that there is something about this sort of meaning that is particularly likely to give rise to implicatures, and thus that our findings in this area are again primarily related to scale-based factors rather than socially or politeness-motivated ones.

Another way in which polarity may play a role (independent of politeness) in the derivation of scalar implicature is by introducing certain presuppositions. Cruse (1986) discusses differences in the scale structure between positive and negative members. He notes that for the interpretation of positive adjectives like *good* the whole scale is relevant while in the case of negative adjectives like *bad*, the underlying question is to put the predicate on the “badness scale.” For this reason, it could be the case that when a speaker utters a sentence like *The movie was bad*, listeners are more likely to derive the inference that the movie was bad but not terrible. In effect, the presuppositions of the predicate may constrain the alternatives available for scalar implicature. Since positive members do not tend to introduce a presupposition it is less clear which alternatives are relevant and therefore hearers may be less likely to derive a scalar implicature.

We conclude that there is some evidence that polarity plays a role in implicature computation but the specific contributions to scalar implicature and negative strengthening need to be determined by further experimental research. It has to be kept in mind that our study was purely correlational (in contrast to other studies demonstrating politeness effects in scalar implicature computation such as Bonnefon et al., 2009). To discover effects of politeness, test sentences may have to be embedded within a rich conversational context in future studies and politeness may have to be manipulated directly in the experimental setup.

### 3.5. Methodological issues

In a commentary, McNally (2017) argues that the methods used by van Tiel et al. were too crude to (i) detect certain implicatures and (ii) detect effects of the parameters they tested. Essentially, the problem McNally discusses is that adjectives are polysemous and in the absence of a context participants may construct the meaning on a fly and not think of the intended pair as scale-mates. This criticism also applies to the current study and it stresses the need to present test sentences within a conversational context. Our investigation particularly motivates further research into the impact of scale structure on implicature derivation. Yet investigating how a large variety of scales behave within an enriched communicative context has to be left to future research. One experimental paradigm which might be useful for this endeavor is the action-based task by Gotzner and Benz (2018), and its interactive version (Benz et al., 2018b), which has been implemented for the quantifier *some* and the connective *or* (Benz and Gotzner, 2017). The advantage of this paradigm is that utterances are embedded in a communicative situation and candidate readings are made relevant. In conclusion, it is vital to move to an experimental paradigm that introduces a context with respect to which statements involving scalar terms should be interpreted.

## 4. Conclusions

Our research revealed an interaction between scalar implicature and negative strengthening, which are based on distinct conversational principles, the Q and R principle, respectively (Horn, 1989; Levinson, 2000). Specifically, participants were less likely to endorse a scalar implicature when they applied negative strengthening to the stronger scale-mate. Importantly, we observed that gradable adjectives do not generally lead to low rates of scalar implicature. Rather, different factors determine which inferences arise with negative strengthening being one of them.

We showed that the most important predictors explaining differences across triggers was the underlying scale structure of the adjectives we tested (in particular boundedness, semantic distance, extremeness, and polarity). Thus far, insights concerning the semantics of scales have not been well integrated into theories of scalar implicature and negative strengthening. Our findings highlight that adjectives with different scale structure give rise to distinct inference patterns. For this reason, we propose that the semantics of different scales should be a central aspect of study in theories of implicature.

## Author contributions

NG has carried out the experiments, analyzed the data, and written the first draft of the manuscript. NG, SS, and AB have all contributed to designing the experiments, annotating the factors and editing subsequent drafts of the manuscript.

### Conflict of interest statement

The authors declare that the research was conducted in the absence of any commercial or financial relationships that could be construed as a potential conflict of interest.

## Footnotes

^1^For example, Levinson (2000) assumes that Q and I implicatures are additive if an utterance triggers both kinds of inferences but if the two interpretations stand in conflict, the Q implicature wins over the I implicature.

^2^Note that the term “scale” is used in two distinct ways in this context, referring either to the scale of lexical alternatives involved in scalar implicature calculation or to the measurement scale that provides the semantic content of individual lexical items. When it is necessary to avoid confusion, we will uses the terms “lexical scale” and “measurement scale” to distinguish these.

^3^The original list contained 71 pairs, but the pair *content/unhappy* was excluded from further analyses on the basis of diagnostics showing that the two terms are not on the same scale, but rather have opposite polarity.

^4^We also computed the semantic similarity of the weak and strong term with latent semantic analysis (Landauer, 2006). For this analysis, we used the tool provided at http://lsa.colorado.edu/ (pairwise comparison). Both the cloze task and the LSA analysis were intended as a measure of the association strength between the weaker and stronger alternative. However, since LSA values were not significant predictors for variability in scalar implicature and negative strengthening rates, we only kept the measure of cloze probability in the final statistical models reported in section 3.2.

^5^The initial model used relative weak terms as the reference level but we also computed a model with minimum standard adjectives as the reference level. Maximum standard adjectives yielded lower SI rates than minimum standard ones (*p* < 0.01) while there was no difference between minimum and relative adjectives (*p* = 0.62).

^6^Again we computed a second model with minimum standard adjectives as the reference level. Maximum standard adjectives yielded higher NegS rates than minimum standard ones (*p* < 0.05) while there was no difference between minimum standard and relative adjectives (*p* = 0.36).
