# Supplementary material for: Scalar Diversity, Negative Strengthening, and Adjectival Semantics
Source: Front Psychol. 2018 Sep 12;9:1659. doi: 10.3389/fpsyg.2018.01659 (PMC6146139; doi:10.3389/fpsyg.2018.01659)
Supplement: Supplementary file 1 [file Data_Sheet_1.pdf]

Appendix

Table A1: Adjective pairs with annotated scale structure and mean rating in scalar implicature (SI) and negative strengthening task (NegS)

adjective pair	upper	lower	polarity	extreme	SI	NegS	dist
attractive/stunning	NB	rel	pos	extreme	0.08	0.6	6.03
drunk/plastered	NB	min	pos	extreme	0.11	0.84	5.32
thin/skinny	NB	rel	neg	extreme	0.11	0.9	3.42
transparent/crystal clear	B	max	pos	extreme	0.11	0.73	4.63
ajar/open	B	max	pos	non	0.11	0.78	3.29
big/enormous	NB	rel	pos	extreme	0.14	0.75	6
dirty/filthy	NB	min	pos	extreme	0.14	0.59	5.39
fat/obese	NB	rel	pos	extreme	0.16	0.72	5.5
tasty/delicious	NB	rel	pos	extreme	0.16	0.88	4.9
damaged/broken	B	min	neg	non	0.16	0.82	3.98
special/unique	B	rel	pos	extreme	0.17	0.77	4.58
snug/tight	NB	rel	neg	non	0.18	0.85	3.39
happy/delighted	NB	rel	pos	extreme	0.19	0.85	5.37
intelligent/brilliant	NB	rel	pos	extreme	0.19	0.6	4.9
pretty/gorgeous	NB	rel	pos	extreme	0.19	0.48	5.87
ugly/hideous	NB	rel	neg	extreme	0.21	0.84	6.05
unkind/nasty	NB	rel	neg	extreme	0.21	0.57	5.6
dry/parched	B	max	neg	extreme	0.22	0.79	5.29
large/gigantic	NB	rel	pos	extreme	0.22	0.74	6.14
content/happy	NB	rel	pos	non	0.22	0.78	4.09
small /tiny	NB	rel	neg	extreme	0.24	0.84	4.53
wet/soaked	NB	min	pos	extreme	0.24	0.44	5.5
calm/unflappable	B	rel	pos	extreme	0.25	0.45	4.85
sickish/sick	NB	min	pos	non	0.25	0.68	4.11
clean/spottless	B	max	neg	extreme	0.27	0.75	5.89
funny/hilarious	NB	rel	pos	extreme	0.27	0.7	5.58
light/white	B	rel	pos	non	0.27	0.33	4.21
rainy/pouring	NB	min	pos	extreme	0.29	0.6	5.93
unhappy/miserable	NB	rel	neg	extreme	0.29	0.69	6.23
tired/exhausted	NB	min	neg	extreme	0.3	0.65	5.16
damp /wet	NB	min	pos	non	0.3	0.65	4.66
poor/destitute	NB	rel	neg	extreme	0.31	0.73	5.47
old/ancient	NB	rel	pos	extreme	0.32	0.58	5.74
scared/petrified	NB	rel	neg	extreme	0.32	0.71	5.97
silly/idiotic	NB	rel	neg	extreme	0.32	0.65	5.49
chubby/fat	NB	rel	pos	non	0.32	0.65	4.74
loud/defeaning	NB	rel	pos	extreme	0.34	0.48	6.2
hot/scalding	NB	rel	pos	extreme	0.35	0.58	5.79

Table A2: Adjective pairs with annotated scale structure and mean rating in scalar implicature (SI) and negative strengthening task (NegS)

adjective pair	upper	weak	polarity	extreme	SI	NegS	dist
memorable/unforgettable	B	rel	pos	extreme	0.37	0.58	5.28
dark/black	B	rel	neg	non	0.37	0.3	4.26
hungry/starving	NB	rel	neg	extreme	0.38	0.46	5.92
tipsy/drunk	NB	min	pos	non	0.38	0.59	5.39
silly/ridiculous	NB	rel	neg	extreme	0.39	0.71	5.57
faulty/non-functional	B	min	neg	non	0.39	0.63	4.08
cool/cold	NB	rel	neg	non	0.43	0.54	4.92
adequate/good	NB	rel	pos	non	0.44	0.78	4.23
sick /terminally ill	NB	min	pos	extreme	0.45	0.29	6.75
wary/scared	NB	rel	neg	non	0.45	0.5	5.05
bent/twisted	NB	min	pos	extreme	0.46	0.6	4
palatable/delicious	NB	rel	pos	extreme	0.47	0.57	5.88
cold/freezing	NB	rel	neg	extreme	0.49	0.58	5.98
annoyed/angry	NB	rel	neg	non	0.51	0.43	5.56
hard/unsolvable	B	rel	neg	extreme	0.52	0.41	6.16
cleanish/clean	B	max	neg	non	0.55	0.78	4.5
good/perfect	B	rel	pos	extreme	0.58	0.45	6.51
possible/certain	B	min	pos	non	0.58	0.3	6.13
unsettling/horrific	NB	rel	neg	extreme	0.62	0.5	6.1
quiet/inaudible	B	rel	neg	extreme	0.63	0.44	5.18
good/excellent	NB	rel	pos	extreme	0.65	0.41	6.05
mediocre/bad	NB	rel	neg	non	0.7	0.49	4.57
warm/hot	NB	rel	pos	non	0.7	0.41	5.45
allowed/obligatory	B	min	pos	non	0.73	0.39	5.37
rare/extinct	B	rel	neg	non	0.74	0.39	6.26
difficult/impossible	B	rel	neg	extreme	0.76	0.35	6.47
cheap/free	B	rel	neg	non	0.76	0.41	5.54
scarce/unavailable	B	rel	neg	non	0.76	0.58	4.92
satifisfactory/impeccable	B	rel	pos	extreme	0.79	0.5	6.21
likely/certain	B	rel	pos	non	0.79	0.53	5.96
low/depleted	B	rel	neg	non	0.81	0.62	4.97
sleepy/asleep	B	rel	neg	non	0.81	0.44	5.26
